# Markers of Antiseizure Treatment Efficacy in Children: A Review

**DOI:** 10.3390/jcm15114194

**Published:** 2026-05-29

**Authors:** Martyna Śliwińska, Anna Rakuś-Kwiatosz, Magdalena Chrościńska-Krawczyk

**Affiliations:** 1 Department of Neonate and Infant Pathology, Medical University of Lublin, 20-093 Lublin, Poland; anna.rakus-kwiatosz@umlub.pl; 2 Department of Pediatric Neurology, Medical University of Lublin, 20-093 Lublin, Poland; magdalena.chroscinska-krawczyk@umlub.pl

**Keywords:** epilepsy, seizures, markers, antiseizure treatment efficacy

## Abstract

**Introduction**: Epilepsy is a chronic brain disorder causing recurrent seizures as a result of abnormal electrical brain activity. Approximately 70 million people worldwide suffer from it and need antiseizure treatment. The diagnosis and assessment of the effectiveness of the therapy are mainly based on symptoms described by a witness or a patient, which is a method of low reliability. The review aims to evaluate available markers of antiseizure drugs’ efficacy, highlighting those that could support the proper course of treatment. **Results**: Comparing the available studies was very challenging. There were significant differences across the groups enrolled in the studies regarding the type of epilepsy included, the patient inclusion criteria, and the total number of patients. Moreover, most of the trials have been conducted on adults, not on children. **Conclusions**: Drug selection and their dosage are mainly based on the relationship between patients and their caregivers. Additionally, electroencephalography (EEG) and antiseizure medication (ASM) concentrations are assessed, but according to many studies, none of the applied methods are sufficient for the diagnostic and therapeutic process to be based on.

## 1. Introduction

Epilepsy is a chronic neurological disease mainly characterized by spontaneous and recurrent seizures [1,2]. Repeated attacks cause brain cell hypoxia, apoptosis, neuronal loss, and abnormal synaptic function that result in varying degrees of developmental delay, learning, behavioral difficulties, and intellectual disability [3]. Epilepsy affects about 1% of the general population, which means approximately 70 million people worldwide suffer from it [1,4,5]. The etiology of the disorder is broad. It has been noticed to be associated with cortical developmental disorders, nervous system infections, tumors, trauma, cerebrovascular diseases, neurodegeneration, genetic and metabolic diseases, and others [5].

The diagnosis is most often clinical and based on the symptoms described by the witnesses or the patient [6,7]. It is usually confirmed by neuroimaging studies or electroencephalography (EEG) [8]. Therefore, it is very easy to make a mistake. In a study of P Uldall et al., 87 out of 223 children (39%) admitted due to drug-resistant paroxysmal events were eventually found not to have epilepsy [8]. Moreover, 35 out of those 87 patients were treated with antiseizure medications (ASMs) at the time of admission [8]. Incorrect diagnosis leads to many problems, such as years of improper medical treatment, sometimes unbearable ASM side effects, lifestyle limitations, and, most importantly, epilepsy stigma [6,9,10]. Moreover, it deteriorates patients’ physical and mental health, builds an economic burden on the patients’ families, and affects society as a whole [1]. Unfortunately, misdiagnosis is not rare [6].

It is believed that up to 30% of epileptic patients have drug-resistant epilepsy (DRE) [1,2,4,5,6,11,12,13]. It is defined as “a failure of adequate trials of two tolerated, appropriately chosen and used ASMs schedules (whether as monotherapies or in combination) to achieve sustained seizure freedom” [14].

Currently, the disease burden and evaluation of the effectiveness of ASMs are measured by the reports from the patients or their caregivers, which is a very untrustworthy method [6,15]. It records only seizures that the person is aware of and is almost useless for people who are unable to communicate their attacks [6]. Moreover, a patient’s memory of a seizure may be impaired as a result of the seizure itself [16]. It is worth emphasizing that almost 30% of epileptic attacks in children are associated with sleep [17].

Blood tests capable of identifying epilepsy, ASM efficacy, and seizure burden would be a more reliable option [6]. Therefore, there is an urgent need to develop reliable biomarkers that would help predict disease progression and treatment response [5,6]. Efficacy markers should be measurable, objective indicators such as biological, electroencephalographic, imaging, or genetic markers that evaluate the therapeutic effect of a drug. There is no known fully approved, specific biomarker for ASM efficacy, disease severity, or neurocognitive comorbidity [18]. Indicators of disease activity, like NT-proBNP for heart insufficiency or HBA1c for diabetes, would facilitate management of epilepsy in the future [6].

The Food and Drug Administration and other agencies accept a 50% responder rate and seizure reduction as important indicators of response in ASM trials [19]. The ILEA, in their final report, recognized that the measurement of effects of pharmacotherapy, the composite effect of epilepsy, and its treatment on patients’ quality of life were important [19,20]. According to the caregivers, resolution of seizures is the most important measure of ASM efficacy, and reduction in seizure frequency is the second most important result of treatment [19]. However, caregivers who evaluated their child’s epilepsy as controlled did not all report seizure freedom [19]. The meaning of control changed depending on the previous course of the disease—patients with drug-resistant seizures were considered to be controlled even though they had three times more attacks than newly diagnosed patients [19].

Currently, many scientific studies focus on investigating biomarkers that allow prediction or assessment of the effectiveness or resistance to ASM treatment. At present, the main candidates include electrophysiological markers, neuroimaging, and molecular and biochemical markers.

The purpose of this review was to evaluate the available markers of antiseizure drug efficacy and assess whether any of them are sufficient.

We have searched the PubMed database by combining the following MESH and free terms: “response to antiepileptic drugs”, “epilepsy”, “markers”, “seizures”, “antiepileptic drug*”, “efficacy”.

## 2. Proposed Markers of Antiseizure Treatment Efficacy

**Electrophysiological markers**
**Electroencephalography**

EEG is one of the main, non-invasive diagnostic methods allowing for assessing bioelectric brain activity [21]. An 11-year retrospective study on the clinical utility of EEG in the pediatric population demonstrated that sleep EEG is superior to awake EEG in detecting epileptic abnormalities. The majority of abnormalities are seen within the first 20 min of recording, but increasing the EEG duration to 30 min significantly increases the utility of EEGs, and almost half of the recorded EEGs showed some kind of abnormality [21]. Another author noticed that ambulatory EEG recordings among children are clinically useful in two-thirds of cases [22]. In the study, encompassing 128 patients with epilepsy, the authors evaluated response to the ASM treatment by the EEG [23]. It was observed that abnormal EEG is an effective element in the assessment of negative therapeutic response, because it significantly correlated with poor response to treatment [23]. In a study by Braathen G. et al., abnormal spike-waves in EEG one year after treatment and 3 Hz spike-waves during the first 6 months of therapy were such elements [24]. Moreover, the systematic review by Lotte Noorlag et al. demonstrated that pathological high-frequency oscillations (HFOs) in scalp EEG have localizing, diagnostic, and prognostic significance, especially in children with epilepsy [25]. On the other hand, in a systematic review conducted by Claire Gunawan et al., a reduced epileptiform discharge burden was revealed as a result of ASMs therapy in genetic generalized epilepsy. It was associated with improved seizure control and cognitive outcomes [26]. Different suppression rates of interictal epileptiform activity were noticed on a follow-up EEG after initiation of therapy with ASMs [27]. A multicenter study performed by Beril Dilber et al. evaluated the effectiveness of ASMs for seizure outcome with respect to the spike-wave index (SWI) on serial EEG recordings. SWI is a quantitative scoring system for EEG calculated during NREM sleep in children with epilepsy. It revealed that SWI may be a valuable indicator of treatment response in children diagnosed with self-limited epilepsy with centrotemporal spikes [28].

**Neuroimaging markers**
**Neurostimulation system**

The RNS System (responsive neurostimulation) is an additional treatment for people with epilepsy [15]. In the study conducted on adults with medically intractable partial onset seizures with two seizure foci, the first week after starting a new ASM, prediction of clinical efficacy with a positive predictive value of 100% and a negative predictive value of 78.6% was observed with the RNS system [15]. The authors noticed that it may provide an objective assessment of cortical excitability and response to ASMs [15]. In the research comparing patients before and after implementing ASMs by the RNS system, a greater reduction in long episodes—often electrographic seizures in the first week for clinically efficacious compared to inefficacious medications—was reported [29]. The lack of long episodes in the first week was highly predictive of ASM efficacy [29].

**Transcranial magnetic stimulation**

Transcranial magnetic stimulation (TMS) was first assessed in 1985 by Barker et al. as a non-invasive technique that may directly stimulate the human motor cortex [30]. In several studies, diminished cortical excitability after the introduction of ASM in patients with epilepsy has been observed [30]. Ziemann et al. revealed that voltage-dependent sodium and calcium channel blockers raise resting motor thresholds, and GABAergic ASM enhances intracortical inhibition [30]. The authors revealed no differences in cortical excitability between children with generalized and focal epilepsy at baseline [30]. Resting motor thresholds raised and cortical silent periods diminished under treatment with sodium valproate [30]. Andreasson et al.’s findings support the potential role of TMS as a biomarker of drug response [31]. However, authors of the retrospective study of long-interval paired pulse TMS based on data collected in four different centers do not support previous promising findings about the utility of TMS as a biomarker [32]. No significant differences in long-interval intracortical inhibition between participants with refractory genetic generalized epilepsy, patients with refractory focal epilepsy, and healthy controls were noticed [32].

**Functional magnetic resonance imaging**

Recent studies have noticed that changes in brain activity induce amplitudes of fluctuations of the blood oxygen level-dependent signals [16,33]. In resting-state functional magnetic resonance imaging (fMRI), these changes may be observed [33]. Yun Jeong Leea et al. reported that combining simultaneous electroencephalography and functional magnetic resonance imaging (EEG-fMRI) can be used to detect the epileptogenic foci [34]. In another study, authors claimed that patients with increased brain activity in the Broadmann area (BA17) in the resting state may have poor seizure control with ASM [33]. Moreover, it was revealed that activated BA17 stimulates many other parts of the brain [33]. Previous research on visually induced seizures has revealed that epileptic discharges of BA17 can create dysesthetic symptoms [33].

**Molecular markers**
**MicroRNAs**

In the study conducted on children with temporal lobe epilepsy (TLE), the serum expression level of miR-15a-5p was decreased in patients with TLE compared to the healthy group [35]. In another study on adults with TLE, an elevated level of miR-34a-5p and a decreased level of miR-106b-5p, -130a-3p, -146a-5p, and -451a in blood samples of DRE TLE subjects was found [36]. Ya Liu et al. revealed that the level of mir-155 in serum exosomes of the children’s epilepsy group was higher, as compared to the healthy control group, and the relative expression of miR-155 in serum exosomes in epileptic children was correlated with the course of the disease and the degree of abnormal EEG [37]. In a meta-analysis by Yi Wang et al., the expression level of miR-155 in the brain tissue and serum of epilepsy patients increased compared to healthy controls [38]. Zhao et al. investigated the serum levels of miR-106b in two groups of children with epilepsy: one treated with sodium valproate and the other treated with sodium valproate and levetiracetam. They revealed that before therapy, the expression of miR-106b for both groups was higher than after treatment. Moreover, in the group treated with sodium valproate and levetiracetam, miR-106b expression was lower than in the sodium valproate group following treatment [39]. In a study of Xiaofeng Wang et al., plasma miRNA-134 level was up-regulated when compared with the level in healthy controls, and down-regulated after treatment of valproic acid in new-onset epilepsy patients [40].

**Biochemical markers**
**Therapeutic drug monitoring**

Therapeutic drug monitoring (TDM) is performed to measure the ASMs and their metabolites concentration in plasma and other biological fluids in relation to the timing of the latest dose administered [41,42,43]. The technology of quantifying drug concentrations in plasma allows us to study the relationship between drug dosage, its concentration in body fluids, and pharmacologic effects [44]. Moreover, saliva-based measurements are gaining increasing popularity due to their advantages [43]. Its use has been demonstrated in studies for specific drugs, commonly valproic acid, levetiracetam, lamotrigine, carbamazepine, and others [43,45].

Many times, incorrect TDM reveals why patients do not respond satisfactorily to a particular dose [44]. On the other hand, in a study conducted by Yunxiu Huang et al., serum valproic acid concentration in more than half (52.5%) of patients with refractory epilepsy µg/patients was between 50 and 100 mL, which is believed to be a therapeutic level [46]. The study conducted by Meral Demir et al. emphasizes the vital role of TDM in a personalized treatment [20]. It should be a very important indicator, because the same drug dose administered to a different patient may result in a different serum level [20]. Therefore, the same ASM dosage is effective in some patients but not in others [44]. This is probably associated with the pharmacokinetic characteristics of ASMs [20].

**Gas chromatography-mass spectrometry**

It is suggested that drug resistance in epilepsy may be connected with a patient’s metabolic profile [47]. Elevated levels of L-glutamine, pyruvic acid, caprylic acid, L-serine, oxalic acid, and aspartic acid were noticed in drug-resistant cases, while palmitic acid was lower [47]. In a study by Tomoyuki Akiyama et al., elevated levels of 2-aminobutyric acid, 2-ketoisocaproic acid, 4-hydroxyproline, acetylglycine, methionine, *N*-acetylserine, and serine were detected in the cerebrospinal fluid of pediatric patients with epilepsy receiving treatment with valproic acid [48].

**Neuropeptides**

Neuropeptides are modulators of classical neurotransmitters [49].

One of these is orexin. In human and in vivo studies, levels of orexins have been connected with epilepsy and sleep disorders [50]. In a study conducted by Samzadeh et al., it was observed that cerebrospinal fluid orexin levels were decreased after recurrent generalized tonic–clonic seizures [50]. However, in a study conducted by Mutluay Arslan et al., which enrolled epilepsy patients with seizures in the last 24 h, epilepsy patients in remission, and healthy controls, no statistically significant difference in serum orexin levels was revealed [50]. Authors highlighted that these differences may reflect age-related variations and orexins’ role in epilepsy pathophysiology [50].

Another peptide explored in epileptic patients is ghrelin. Significant rise in the serum, urine, and saliva levels of ghrelin and des-acyl form of ghrelin among 30 pediatric patients with idiopathic generalized epilepsy during valproate therapy was observed [51]. In another study, ghrelin levels were diminished in young epileptic prepubertal children during treatment with carbamazepine and valproic acid when compared to a healthy group [52].

Nesfatin-1 is a neuropeptide expressed in many areas of the brain [53]. The connection of this hormone with epilepsy has been studied many times. Serum nesfatin-1 levels were elevated in newly diagnosed primary generalized epilepsy patients in relation to controls [53]. However, this rise was reduced via ASM therapy, but the level still remained higher than in the control group [53].

**Other proteins**

Many other proteins were investigated as serum biomarkers of DRE [5]. One of them is glutamate cysteine ligase catalytic subunit (GCLC), which is down-regulated in the serum of patients with DRE [5]. PRDX6 was identified to be decreased in patients with DRE compared with controls [5]. However, in epileptic rats, it was upregulated [5]. The anti-inflammatory endogenous protein membrane-associated protein A1 (ANXA1) is likely involved in the progression of neurological disorders [5]. The authors’ results suggested that the serum level is reduced in DRE patients [5]. S100A6 (calcineurin) is a calcium-binding protein believed to be associated with calcium homeostasis and neuronal degeneration [5]. It was observed to be diminished in the serum of patients with DRE [5].

α-Synuclein is one of the most frequently occurring proteins in the central nervous system [6]. A study conducted on children with epilepsy revealed that serum α-synuclein levels were raised in the epilepsy group and correlated with the disease severity [6]. Moreover, exosomal α-synuclein levels were associated with serum IL-6 levels in children with epilepsy and patients with acquired demyelinating disorders of the CNS [6].

In previously performed studies, a relationship between haptoglobin and epilepsy was proposed [54]. Biomarkers, such as haptoglobin, IFN-γ, and IL-1β, may help in early diagnosis of refractory epilepsy, monitoring the disease progression and treatment response [54].

Elevated levels of pro-inflammatory cytokines such as interleukin-2 (IL-2), tumor necrosis factor-α (TNF-α), and interferon-α (INF-α) have been revealed in the blood of young patients with the diagnosis of West syndrome prior to treatment [55]. It was observed that adrenocorticotropic hormone (ACTH) used in the treatment of infantile spasms significantly reduced levels of interleukin-6 (IL-6), interferon-γ (IFN-γ), and macrophage inflammatory protein-1 α (MIP-1α) [55]. However, the exact antiepileptic mechanism of the action of ACTH is still unclear [55]. Decreased levels of interleukin-10 (IL-10) and IFN-γ under levetiracetam treatment were observed [55]. However, the concentration of TNF-α was raised and the concentration of MIP-1α was diminished in the group receiving valproic acid [55]. These findings suggest that ASMs interact with the immunological system. Steinborn et al. analyzed changes in the levels of certain cytokines (interleukin-1ß, IL-2, IL-6, TNF-α) in patients with newly diagnosed generalized epilepsy treated with valproic acid [55]. They revealed a decreased level of IL-6 after 4–6 months of valproic acid therapy [55]. This may be a common antiseizure mechanism of action of valproic acid and ACTH [55].

It was revealed that interleukin-1 receptor type 1 (IL-1R1) and toll-like receptor 4 (TLR4) increase in microglia, astrocytes, and neurons during cell damage and seizure [56]. Raised high mobility group box 1 protein (HMGB-1), TLR4, TNF-1, IL-1R1, and IL-1 levels in the severe epilepsy group compared to mild epilepsy and control groups were noticed [56]. Moreover, patients with DRE had elevated HMGB-1 levels [13,56]. On the other hand, Yulia S. Panina et al. noticed a lower concentration of brain-derived neurotrophic factor (BDNF), TNFa, and HMGB1 proteins in the group of patients with temporal lobe epilepsy compared with the control group [11]. In a study performed by Min Zhu, MD et al. evaluated 180 children with new-onset epilepsy and 40 healthy children; serum concentrations of HMGB1 were elevated in patients with epilepsy [57]. In animal models, an anti-HMGB1 monoclonal antibody had a protective effect on neuronal apoptosis and epileptogenesis by inhibition of HMGB1 release [1]. Moreover, glycyrrhizin—an HMGB1 inhibitor—has antiepileptic properties and was protective for the neurons in animal models of epilepsy [12].

Strauss KI et al. assessed cytokines in resected hippocampus, entorhinal cortex, and temporal cortex samples obtained from 58 medically refractory mesial temporal lobe epilepsy patients and 4 nonepileptic neurosurgical patients [54]. The analysis revealed that higher IL-1β levels were noticed in non-epileptic brain areas than epileptic brain areas, thus suggesting neuroprotective roles of IL-1β [54]. In a study of adolescent females with refractory epilepsy, patients were treated with an IL-1 antagonist and with an IL-1B antibody during a nonconvulsive status epilepticus, which led to full resolution of clinical seizures [1]. Interestingly, the authors of another study noticed that serum levels of some cytokines: IFN-γ, IL-1ß, IL-6, and IL-10 showed a negative correlation with the age of the epilepsy onset in pediatric patients, indicating that the pediatric population presented higher inflammatory responses to afebrile seizure attacks [6]. In their study, a correlation between serum IL-1β levels and the numbers of ASMs being used by patients was observed within 48 h after afebrile seizure episodes; thus, the increase in serum IL-1β may be linked with drug resistance, current epileptogenesis, or neuroinflammation [6].

It was observed that drug-free patients with epilepsy had elevated levels of plasma prostaglandin E2 compared to healthy cases [58]. Moreover, responders and non-responders receiving phenytoin or carbamazepine monotherapy presented higher levels than healthy subjects [58]. Valproate responders had a decreased level of prostaglandin E2 compared to non-responders and the drug-free group [58]. Authors have suggested that valproate-mediated prostaglandin E2 reduction may be connected with drug efficacy [58]. Schlichtiger et al. assessed the efficacy of the selective COX-2 inhibitor-celecoxib administration in epileptic rats [14]. They noticed that celecoxib diminished P-gp expression and seizure frequency in both responders and non-responders, suggesting the potential fundamental role of COX-2 inhibitors in increasing ASM efficacy [58]. However, Chitra Rawat et al. observed no difference in the plasma prostaglandin levels between the responder and non-responder groups treated with carbamazepine and phenytoin [58]. Both had higher plasma prostaglandin E2 levels than healthy controls [58].

BDNF is a small protein encoded by the human BDNF gene [20]. It plays a crucial role in the neuronal survival, growth, and differentiation of new neurons and synapses during brain development [20]. Meral Demir et al. performed a study that enrolled patients treated with new ASMs and assessed their BDNF serum level [20]. They noticed elevated BDNF levels in the group of patients treated with ASM with efficient, safe, and appropriate doses and decreased levels of BDNF in the resistant cases [20]. On the other hand, a previously conducted study revealed reduced BDNF mRNA in the cingulate cortex, hippocampus, and thalamus in patients under treatment with phenobarbital, valproate, and phenytoin [11]. According to another study that assessed plasma and serum concentrations of proteins associated with neuroinflammation and neurodegeneration in patients with TLE, lower plasma BDNF levels in adult patients with TLE compared to controls may be triggered by the use of ASMs [11]. It reflects the duration of TLE and may indicate the suppression of neuroplasticity and neurogenesis in TLE patients [11].

## 3. Discussion

Epilepsy is a neurological disease characterized by a predisposition to generate epileptic seizures [32]. The diagnosis is based on clinical history, often supported by epileptiform discharges on EEGs [32]. However, abnormal brainwave patterns on EEGs do not always occur during a short recording [32].

The goal of our review was to find the proposed markers of antiseizure treatment efficacy in the literature.

Our analysis revealed that not many studies concerning the aforementioned issue have been conducted in children so far. Moreover, there were differences across the groups enrolled in the studies concerning the type of epilepsy, the patient inclusion criteria, and the number of patients. Therefore, comparing these studies was very challenging.

TDM was assessed for a number of ASMs to evaluate optimal therapy for individuals [20]. The initial studies were conducted in the fifties and early sixties of the 20th century [20]. Monitoring the effectiveness of antiepileptic treatment is compulsory. Follow-ups are crucial, especially in the youngest patients who gain weight and need changes to the dosage. Regular hospital visits may be difficult, especially for children with disabilities, so the collection of dried blood spot samples at home has been investigated [41]. Moreover, saliva-based TDM, which is becoming a more common form of TDM, is minimally invasive and does not require needle puncture, which is particularly important in the pediatric population [43]. TDM is commonly used by neurologists to correlate the occurrence of seizures with an adequate dose of an ASM, which we support [20]. However, TDM cannot be the only indicator in some cases, because the same dosage of the ASM may cause different serum concentrations, and some patients with therapeutic drug concentrations may still exhibit symptoms [20,46].

EEG helps to identify various neurological conditions and to differentiate the episodes simulating epilepsy [21]. The diagnostic sensitivity of routine EEG for epilepsy is estimated at 17% in adults and at 58% in children [32]. Interestingly, among children with epilepsy, the EEG is frequently normal, and epileptiform EEG discharges may be observed in up to 5% of children without epilepsy [8]. Sensitivity may be enhanced by extending recording time or by implementing methods such as sleep deprivation, hyperventilation, or photic stimulation [32]. The majority of the abnormalities on routine EEGs are noticed within the first 20 min, but even increasing the duration to 30 min enhances its efficacy [21]. The ILEA recommends at least 30 min of EEG recording [21]. To increase the usefulness of routine EEG recording, it should be repeated [22]. The ILEA recommends long-term EEG monitoring to assess the effects of drug interventions [22]. Moreover, according to ILEA, routine and sleep EEG have an established role in clinical diagnosis of epilepsy; however, long-term video-electroencephalographic monitoring should be used to differentiate between epileptic and non-epileptic events in adults and children when seizures remain uncontrolled despite appropriate treatment [59]. The results of a systematic review conducted by Raras Windaswara et al. also confirm the aforementioned statement [60]. Moreover, the use of artificial intelligence in the interpretation of EEG may become an indispensable part of the diagnostic procedure in the future, but more studies are needed to assess it [60]. Furthermore, a twenty-four-hour ambulatory EEG may be a very useful method for measuring epileptiform discharges in some cases. It is 75% cheaper and better tolerated by patients [26]. We fully support the use of these methods, but we are aware of their limitations [27].

The RNS system enables the detection of specific patterns characteristic of the onset of an electrographic seizure and epileptiform discharges [15]. The device is placed within the cranium and connected to two leads that are located according to the patient’s seizure focus [15]. Each lead contains four sensing and stimulating electrodes [15]. After noticing the event, it responds by providing short bursts of electrical stimulation [15]. Recent trials support the clinical use of RNS as an epilepsy biomarker. The utility of ambulatory intracranial electrocorticographic provided by RNS to assess therapeutic responses in focal epilepsy within 1–2 weeks of starting a new ASMs was reported [29]. Cortical excitability can also be measured in a non-invasive way using TMS [31,32]. TMS-evoked EEG responses may be potential biomarkers of a disease and treatment response [61]. In a review performed in 2022, assessing the utility of TMS combined with EEG and TMS-EEG, inconsistent findings on the validity of TMS-EEG as an epilepsy biomarker were reported [61]. Functional magnetic resonance imaging (fMRI) also allows us to detect changes in brain activity [16]. Studies combining fMRI and simultaneous electroencephalography have been performed. However, performing EEG-fMRI in pediatric patients is challenging because of the lack of cooperation and the need for sedation in the youngest patients [34]. Yun Jeong Leea et al. have noticed that this neuroimaging technique can provide additional information about the epileptogenic zone in children with focal epilepsy [34]. High brain activity in BA17 observed in uncontrolled seizures in TMS might be associated with disturbance in the brain activation and inhibition processes [33]. It suggests that the drug-resistant epileptogenic networks not only disturb ASMs from reaching their target but also dysregulate brain activity and destroy the balance of neuronal activation and inhibition [33]. Detection by f(MRI) of changes in brain activity, which causes different spontaneous amplitudes of low-frequency fluctuations of the blood oxygen level-dependent signals, may be a promising biomarker of ASMs treatment efficacy [33]. However, further studies are needed to assess the true role of these biomarkers.

MiRNAs are small noncoding RNAs that regulate multiple processes at the posttranscriptional level [35]. Obtaining miRNA levels in blood is an easy, quick, accessible, and slightly invasive method [8]. Overexpression of miR-15a-5p extends cell lifespan and inhibits apoptosis, which may be connected with the progression of children’s TLE [35]. It has been suggested that in the advanced stages of the disease, the decrease in some miRNAs may be responsible for drug resistance [36]. The authors highlighted that the differences in the amount of different miRNAs found in their analysis could be influenced by the age of the patients: the presence of some miRNAs could be connected with their role in the growth of the organism [36]. In another review article, the possible utility of miRNAs was described [7,62]. Beata Rzepka-Migut and Justyna Paprocka suggested that different miRNAs could be biomarkers of epilepsy, drug resistance, surgical prognosis, and effectiveness of therapy [8]. In a study by Liu Y et al., the expression level of exosomal miR-155 elevates with the disease severity, showing its significance for the prognosis evaluation of children. Moreover, its level was related to the degree of abnormal EEG in children, so it could be used as a new marker for assessment of the severity of epilepsy [37]. The expression of other mi-RNA-miR-106b in children after treatment with sodium valproate and levetiracetam gradually decreased with the improvement of the disease, showing that miR-106b may be a potential prognostic marker for epileptic children after treatment [39]. According to the results of the study conducted by Xiaofeng Wang et al., the upregulation of plasma miRNA-134 levels may serve as a biomarker in epileptic patients, and the downregulation of plasma miRNA-134 levels is correlated with the effectiveness of ASM treatment [40].

Elevated glutamine levels in astrocytes detected in GC-MS testing may result in increased glutamic acid release into the extracellular space [47]. L-serine is crucial for neuronal differentiation and development, so its higher level in DRE patients may be a compensatory neuroprotective action against refractory seizures [47]. Caprylic acid, a crucial element of the medium-chain triglyceride ketogenic diet, shows acute anticonvulsant effects and intensifies the anticonvulsant effects of valproic acid [47]. Moreover, the results obtained from the GC-MS analysis of the cerebrospinal fluid of pediatric patients with and without epilepsy revealed that valproic acid impacts the levels of amino acids and related metabolites associated with the metabolism of serine, methionine, and leucine [48]. However, future studies on the issue are needed.

Neuropeptides modify neurotransmitter release, regulate their effects at the receptor level, and influence the balance between the inhibition and excitation processes [49]. Orexins take part in energy homeostasis, eating and drinking behaviors, sleeping regulation, analgesia, and cognitive activities [50]. Their receptor antagonists have been found to modulate seizure activity [50]. Ghrelin, with its des-acyl form, is a hormone produced by the P/D1 cells of the stomach [51]. Ghrelin, by activating the GH secretagogs (GHS) receptor type 1a (GHS-R1a), contributes to releasing growth hormone [52]. Through neuropeptide Y (NPY), it affects glucose and insulin metabolism and influences food and energy intake [52]. Ghrelin seems to be dysregulated in patients with epilepsy under ASMs treatment [51]. The decreased serum ghrelin level in epilepsy was explained by its increased consumption when dealing with recurrent seizures [53]. It has been suggested that neuropeptides such as leptin, ghrelin, and NPY modulate epilepsy by reducing the proinflammatory cytokine response [63]. Their probable anticonvulsant and protective role seems to be intriguing [51]. Many researchers described that ghrelin, by enhancing NPY and GABAergic activity, has antiepileptic effects [63]. An alternative mechanism is the action via stimulation of the vagal nerve [63]. Nesfatin-1 is another neuropeptide investigated in epileptic patients, which could be a biomarker of the disease. It is secreted mainly by the stomach cells, but it is also present in the brainstem, midbrain, hypothalamus, amygdala, and cerebellum [64]. It is believed that nesfatin-1 acts via a kind of G-protein-coupled receptor (GPCR), but it has not yet been identified [64]. It is suggested that it may be the same receptor through which ghrelin exerts its effect [64]. Nesfatin-1 may have a neuroprotective effect in epilepsy and ischemic stroke by decreasing oxidative stress [65,66,67]. However, further studies are needed to assess the true role of these biomarkers.

It is suggested that the enhancement of GCLC by phenytoin likely raises hippocampal glutathione levels and protects its cells from oxidative stress damage [5]. The S100 protein family encompasses more than 20 calcium-binding proteins [68]. S100B is mainly localized in astrocytes, but its increased level has also been noticed in different biological fluids in various CNS disorders, such as epilepsy [68]. Carbamazepine, oxcarbazepine, or levetiracetam significantly reduce serum levels of S100B [68]. Interestingly, mice lacking a functional S100B gene have been assessed to be more prone to earlier and more severe seizures than wild-type mice [68]. α-Synuclein probably takes part in synaptic transmission, mitochondrial and synaptic dysfunction, neuronal apoptosis, and calcium homeostasis [6]. Oligomeric forms of α-synuclein are included in the neurodegenerative process and enhance synaptic transmission and diminish long-term potentiation [6]. Haptoglobin is a protein synthesized in the liver that binds to free hemoglobin [54]. Upregulation of plasma haptoglobin in refractory epilepsy seems to be a compensatory mechanism against neuronal injury following seizures [54].

Inflammatory mediators secreted by brain cells during epileptic discharges act as neuromodulators and enhance inflammatory reactions [56]. It is worth emphasizing that inflammation lowers the seizure threshold [56]. Trials have revealed that certain inflammatory molecules affect seizure development, epileptogenesis, and drug resistance [56]. Blood HMGB1 levels were connected with MRI abnormalities known to be associated with a high risk of drug resistance [13]. The available data suggest that elevated serum levels of HMGB1 in drug-resistant vs. drug-responsive patients might reflect the ability to respond or not respond to ASMs [13]. Activation by HMGB1/TLR4 signaling phosphorylation of the N-methyl-D-aspartic acid (NMDA) receptor leads to Ca2+ influx, followed by neuronal cell hyperexcitability and epileptogenesis [1]. Serum levels of HMGB1 might be associated with epilepsy after brain injury, so its level in the blood may help to select patients at risk of developing spontaneous seizures after injury [1]. It seems to be a potentially novel prognostic, diagnostic, and predictive biomarker of DRE, seizure relapse, and response to therapy [1,57]. Some authors have planned to validate HMGB1 isoforms as biomarkers of epilepsy and/or the therapeutic response to ASMs [4]. However, to the best of our knowledge, it has not been assessed in epilepsy clinical studies yet [12].

IL-1 binds to IL1R1 and leads to the activation of nuclear factor ka pa-light-chain-enhancer of activated B cells (NF-kB), which enhances the inflammation processes [1]. The IL-1b/IL-1R1 pathways increase NMDA receptor-mediated Ca2+ influx into neurons and inhibit GABA-mediated Cl-influx, resulting in excitotoxicity and seizure activity [1]. Moreover, increased expression of cyclooxygenase-2 (COX-2) has been noticed in the brain of rodent models after a convulsive challenge, increasing the production of prostaglandins, aggravating seizure severity [58]. It may stimulate the P-glycoprotein (P-gp) at the blood–brain barrier and therefore elevate the efflux of the ASMs into the bloodstream, resulting in poor efficacy [58].

The above-mentioned proteins seem to be very promising epilepsy biomarkers. Previous studies supported the use of TNF-α/sTNFr2 and HMGB1/TLR-4 as early biomarkers of DRE [12]. However, the data presented in the review are only an example of the many studied biomarkers, described as being associated with epilepsy. The discussion on all proposed epilepsy biomarkers exceeds the scope of the review, but we chose to present some of them.

## 4. Conclusions

In conclusion, we have identified a number of potential epilepsy markers. Some of them, such as EEG and TDM, are commonly used in the course of epilepsy patients’ treatment, and based on the available articles, this appears to be justified. Moreover, the studies we cited have shown promising outcomes for the use of many other biomarkers. The results obtained from the RNS system, TMS, and fMRI were evaluated for their potential use as markers of ASM efficacy. Some neuropeptides, like orexin, ghrelin, nesfatin-1, and others, as modulators of neurotransmitters, have shown promising results as well. Moreover, pro- and anti-inflammatory cytokines may also serve as biomarkers in the future. However, most of the reported potential markers require future, clinically reliable studies conducted on children with epilepsy in order to demonstrate their clinical usefulness. The results revealed in the review are only a part of the many evaluated biomarkers that could be associated with epilepsy. Presenting all of them exceeds the scope of the review.

## Data Availability

No new data were created or analyzed in this study.

## References

[1] Zhang S., Chen F., Zhai F., Liang S. (2022). Role of HMGB1/TLR4 and IL-1β/IL-1R1 Signaling Pathways in Epilepsy. Front. Neurol..

[2] Pinzon-Hoyos N., Li Y., McGee M., Poolos N.P., Marchi N., Brewster A.L. (2024). Sex-Specific Complement and Cytokine Imbalances in Drug-Resistant Epilepsy: Biomarkers of Immune Vulnerability [Preprint]. bioRxiv.

[3] Wang Q., Shi X., Li P.-P., Gao L., Zhou Y., Li L., Ye H., Fu X., Li P. (2024). microRNA profilings identify plasma biomarkers and targets associated with pediatric epilepsy patients. Pediatr. Res..

[4] Ravizza T., Terrone G., Salamone A., Frigerio F., Balosso S., Antoine D.J., Vezzani A. (2018). High Mobility Group Box 1 is a novel pathogenic factor and a mechanistic biomarker for epilepsy. Brain Behav. Immun..

[5] Ma M., Cheng Y., Hou X., Li Z., Wang M., Ma B., Cheng Q., Ding Z., Feng H. (2024). Serum biomarkers in patients with drug-resistant epilepsy: A proteomics-based analysis. Front. Neurol..

[6] Banote R.K., Akel S., Zelano J. (2022). Blood biomarkers in epilepsy. Acta Neurol. Scand..

[7] Rzepka-Migut B., Paprocka J. (2021). Prospects and Limitations Related to the Use of MicroRNA as a Biomarker of Epilepsy in Children: A Systematic Review. Life.

[8] Uldall P., Alving J., Hansen L.K., Kibæk M., Buchholt J. (2006). The misdiagnosis of epilepsy in children admitted to a tertiary epilepsy centre with paroxysmal events. Arch. Dis. Child..

[9] Hamrah M.P., Tavirani M.R., Movahedi M., Karvigh S.A. (2020). Identification of Serum Biomarkers for Differentiating Epileptic Seizures from Psychogenic Attacks Using a Proteomic Approach; a Comparative study. Arch. Acad. Emerg. Med..

[10] Wang Y.-Q., Wen Y., Wang M.-M., Zhang Y.-W., Fang Z.-X. (2021). Prolactin levels as a criterion to differentiate between psychogenic non-epileptic seizures and epileptic seizures: A systematic review. Epilepsy Res..

[11] Panina Y.S., Timechko E.E., Usoltseva A.A., Yakovleva K.D., Kantimirova E.A., Dmitrenko D.V. (2023). Biomarkers of Drug Resistance in Temporal Lobe Epilepsy in Adults. Metabolites.

[12] Aguilar-Castillo M.J., Cabezudo-García P., García-Martín G., Lopez-Moreno Y., Estivill-Torrús G., Ciano-Petersen N.L., Oliver-Martos B., Narváez-Pelaez M., Serrano-Castro P.J. (2024). A Systematic Review of the Predictive and Diagnostic Uses of Neuroinflammation Biomarkers for Epileptogenesis. Int. J. Mol. Sci..

[13] Walker L.E., Sills G.J., Jorgensen A., Alapirtti T., Peltola J., Brodie M.J., Marson A.G., Vezzani A., Pirmohamed M. (2022). High-mobility group box 1 as a predictive biomarker for drug-resistant epilepsy: A proof-of-concept study. Epilepsia.

[14] Bronisz E., Cudna A., Wierzbicka A., Kurkowska-Jastrzębska I. (2022). Serum Proteins Associated with Blood–Brain Barrier as Potential Biomarkers for Seizure Prediction. Int. J. Mol. Sci..

[15] Skarpaas T.L., Tcheng T.K., Morrell M.J. (2018). Clinical and electrocorticographic response to antiepileptic drugs in patients treated with responsive stimulation. Epilepsy Behav..

[16] Gascoigne S.J., Waldmann L., Schroeder G.M., Panagiotopoulou M., Blickwedel J., Chowdhury F., Cronie A., Diehl B., Duncan J.S., Falconer J. (2023). A library of quantitative markers of seizure severity. Epilepsia.

[17] Kaciński M., Budziszewska B., Lasoń W., Zając A., Skowronek-Bała B., Leśkiewicz M., Kubik A., Basta-Kaim A. (2012). Level of S100B protein, neuron specific enolase, orexin A, adiponectin and insulin-like growth factor in serum of pediatric patients suffering from sleep disorders with or without epilepsy. Pharmacol. Rep..

[18] Choi J., Kim S.Y., Kim H., Lim B.C., Hwang H., Chae J.H., Kim K.J., Oh S., Kim E.Y., Shin J.-S. (2020). Serum α-synuclein and IL-1β are increased and correlated with measures of disease severity in children with epilepsy: Potential prognostic biomarkers?. BMC Neurol..

[19] Perry M.S., Swint C., Hawley J., Kohler S., Blake S., Rask K., Sladky J., Krawiecki N. (2011). Caregiver measures for seizure control, efficacy, and tolerability of antiepileptic drugs for childhood epilepsy: Results of a preference survey. Epilepsy Behav..

[20] Demir M., Akarsu E.O., Dede H.Ö., Bebek N., Yıldız S.Ö., Baykan B., Akkan A.G. (2020). Investigation of the Roles of New Antiepileptic Drugs and Serum BDNF Levels in Efficacy and Safety Monitoring and Quality of Life: A Clinical Research. Curr. Clin. Pharmacol..

[21] Tekin Orgun L., Arhan E., Aydin K., Rzayeva T., Hirfanoglu T., Serdaroglu A. (2018). What has changed in the utility of pediatric EEG over the last decade?. Turk. J. Med. Sci..

[22] Nagyova R., Horsburgh G., Robertson A., Zuberi S.M. (2019). The clinical utility of ambulatory EEG in childhood. Seizure.

[23] Ghofrani M., Nasehi M.M., Saket S., Mollamohammadi M., Taghdiri M.M., Karimzadeh P., Tonekaboni S.H., Javadzadeh M., Jafari N., Zavehzad A. (2018). Prediction of Response to Treatment in Children with Epilepsy. Iran. J. Child Neurol..

[24] Braathen G., Melander H. (1997). Early discontinuation of treatment in children with uncomplicated epilepsy: A prospective study with a model for prediction of outcome. Epilepsia.

[25] Noorlag L., van Klink N.E., Kobayashi K., Gotman J., Braun K.P., Zijlmans M. (2022). High-frequency oscillations in scalp EEG: A systematic review of methodological choices and clinical findings. Clin. Neurophysiol..

[26] Gunawan C., Seneviratne U., D’SOuza W. (2019). The effect of antiepileptic drugs on epileptiform discharges in genetic generalized epilepsy: A systematic review. Epilepsy Behav..

[27] Libenson M.H., Caravale B. (2001). Do antiepileptic drugs differ in suppressing interictal epileptiform activity in children?. Pediatr. Neurol..

[28] Dilber B., Serdaroğlu E., Kanmaz S., Kılıç B., İpek R., Menderes D.K., Yıldız N., Topçu Y., Arhan E.P., Serdaroğlu A. (2024). A Multicenter Study of Self-Limited Epilepsy with Centrotemporal Spikes: Effectiveness of Antiseizure Medication with Respect to Spike-Wave Index. Pediatr. Neurol..

[29] Quraishi I.H., Mercier M.R., Skarpaas T.L., Hirsch L.J. (2020). Early detection rate changes from a brain-responsive neurostimulation system predict efficacy of newly added antiseizure drugs. Epilepsia.

[30] Andreasson A., Sigurdsson G.V., Pegenius G., Thordstein M., Hallböök T. (2020). Cortical excitability measured with transcranial magnetic stimulation in children with epilepsy before and after antiepileptic drugs. Dev. Med. Child Neurol..

[31] Balestrini S., Sander J.W. (2020). Transcranial magnetic stimulation as a biomarker of treatment response in children with epilepsy. Dev. Med. Child Neurol..

[32] Bauer P.R., Goede A.A.d., Stern W.M., Pawley A.D., Chowdhury F.A., Helling R.M., Bouet R., Kalitzin S.N., Visser G.H., Sisodiya S.M. (2018). Long-interval intracortical inhibition as biomarker for epilepsy: A transcranial magnetic stimulation study. Brain.

[33] Hu Y., Mi X., Xu X., Fang W., Zeng K., Yang M., Li C., Wang S., Li M., Wang X. (2015). The Brain Activity in Brodmann Area 17: A Potential Bio-Marker to Predict Patient Responses to Antiepileptic Drugs. PLoS ONE.

[34] Lee Y.J., Bae H., Byun J.C., Kwon S., Oh S.S., Kim S. (2022). Clinical Usefulness of Simultaneous Electroencephalography and Functional Magnetic Resonance Imaging in Children with Focal Epilepsy. J. Clin. Neurol..

[35] Li N., Pan J., Liu W., Li Y., Li F., Liu M. (2020). MicroRNA-15a-5p serves as a potential biomarker and regulates the viability and apoptosis of hippocampus neuron in children with temporal lobe epilepsy. Diagn. Pathol..

[36] Bertoli G., Fortunato F., Cava C., Manna I., Gallivanone F., Labate A., Panio A., Porro D., Gambardella A. (2024). Serum MicroRNAs as Predictors of Diagnosis and Drug-resistance in Temporal Lobe Epilepsy: A Preliminary Study. Curr. Neuropharmacol..

[37] Çarman K.B., Gazeteci Tekin H., Çavuşoğlu D., Yarar C., Kaplan E., Karademir C.N., Arslantaş D. (2023). Evaluation of MicroRNAs in Pediatric Epilepsy. Turk. Arch. Pediatr..

[38] Wang Y., Chen C., Ya C., Chen J., Lu B., Liu J., Wu Q., Diao L., Liu H. (2025). The levels of miR-155 in epilepsy patients: A meta-analysis. Front. Neurol..

[39] Zhao J., Sang Y., Zhang Y., Zhang D., Chen J., Liu X. (2019). Efficacy of levetiracetam combined with sodium valproate on pediatric epilepsy and its effect on serum miR-106b in children. Exp. Ther. Med..

[40] Wang X., Luo Y., Liu S., Tan L., Wang S., Man R. (2017). MicroRNA-134 plasma levels before and after treatment with valproic acid for epilepsy patients. Oncotarget.

[41] Linder C., Wide K., Walander M., Beck O., Gustafsson L.L., Pohanka A. (2017). Comparison between dried blood spot and plasma sampling for therapeutic drug monitoring of antiepileptic drugs in children with epilepsy: A step towards home sampling. Clin. Biochem..

[42] Paul J., Das P., Das A., Ojha B., Das K., Deb T. (2026). Current Scenarios and Future Perspectives of Therapeutic Drug Monitoring in India: A Narrative Review. Cureus.

[43] Patrick M., Parmiter S., Mahmoud S.H. (2021). Feasibility of Using Oral Fluid for Therapeutic Drug Monitoring of Antiepileptic Drugs. Eur. J. Drug Metab. Pharmacokinet..

[44] Patsalos P.N., Berry D.J., Bourgeois B.F., Cloyd J.C., Glauser T.A., Johannessen S.I., Leppik I.E., Tomson T., Perucca E. (2008). Antiepileptic drugs--best practice guidelines for therapeutic drug monitoring: A position paper by the subcommission on therapeutic drug monitoring, ILAE Commission on Therapeutic Strategies. Epilepsia.

[45] Resztak M., Czyrski A., Sobiak J. (2025). Saliva as a matrix for therapeutic drug monitoring and disease biomarkers in children and adolescents. Pharmacol. Rep..

[46] Huang Y., Zhang Z., Chen L. (2023). Diagnosis and prognosis of serum Fut8 for epilepsy and refractory epilepsy in children. PLoS ONE.

[47] Kong S.T., Lim S.-H., Ching J., Ho P.C.-L. (2025). GC-MS uncovers unique metabolic markers of drug-resistant epilepsy in capillary but not venous dried blood spots. J. Pharm. Biomed. Anal..

[48] Akiyama T., Saigusa D., Hyodo Y., Umeda K., Saijo R., Koshiba S., Kobayashi K. (2020). Metabolic Profiling of the Cerebrospinal Fluid in Pediatric Epilepsy. Acta Med. Okayama.

[49] Clynen E., Swijsen A., Raijmakers M., Hoogland G., Rigo J.-M. (2014). Neuropeptides as targets for the development of anticonvulsant drugs. Mol. Neurobiol..

[50] Arslan M., Üstün C., Coşkun A.N., Çelik H., Yalçın H.N., Karaismailoğlu E., Sertoğlu E., Ünay B. (2024). Can serum orexin levels be used as a marker in childhood epilepsy?. Heliyon.

[51] Marchiò M., Roli L., Giordano C., Caramaschi E., Guerra A., Trenti T., Biagini G. (2018). High plasma levels of ghrelin and des-acyl ghrelin in responders to antiepileptic drugs. Neurology.

[52] Prodam F., Bellone S., Casara G., De Rienzo F., Grassino E.C., Bonsignori I., Demarchi I., Rapa A., Radetti G., Bona G. (2010). Ghrelin levels are reduced in prepubertal epileptic children under treatment with carbamazepine or valproic acid. Epilepsia.

[53] Erkec O.E., Milanlıoğlu A., Komuroglu A.U., Kara M., Huyut Z., Keskin S. (1992). Evaluation of serum ghrelin, nesfatin-1, irisin, and vasoactive intestinal peptide levels in temporal lobe epilepsy patients with and without drug resistance: A cross-sectional study. Rev. Assoc. Med. Bras..

[54] Saengow V.E., Chiangjong W., Khongkhatithum C., Changtong C., Chokchaichamnankit D., Weeraphan C., Kaewboonruang P., Thampratankul L., Manuyakorn W., Hongeng S. (2021). Proteomic analysis reveals plasma haptoglobin, interferon-γ, and interleukin-1β as potential biomarkers of pediatric refractory epilepsy. Brain Dev..

[55] Kaczorowska M., Czekuć-Kryśkiewicz E., Dądalski M., Kotulska K. (2023). Immunological markers of drug resistant epilepsy and its response to immunomodulatory therapy with ACTH in children. Folia Neuropathol..

[56] Kamaşak T., Dilber B., Yaman S.Ö., Durgut B.D., Kurt T., Çoban E., Arslan E.A., Şahin S., Karahan S.C., Cansu A. (2020). HMGB-1, TLR4, IL-1R1, TNF-α, and IL-1β: Novel epilepsy markers?. Epileptic Disord..

[57] Zhu M., Chen J., Guo H., Ding L., Zhang Y., Xu Y. (2018). High Mobility Group Protein B1 (HMGB1) and Interleukin-1β as Prognostic Biomarkers of Epilepsy in Children. J. Child Neurol..

[58] Rawat C., Shivangi, Kushwaha S., Sharma S., Srivastava A.K., Kukreti R. (2020). Altered plasma prostaglandin E2 levels in epilepsy and in response to antiepileptic drug monotherapy. Prostaglandins Leukot. Essent. Fat. Acids.

[59] Peltola M.E., Leitinger M., Halford J.J., Vinayan K.P., Kobayashi K., Pressler R.M., Mindruta I., Mayor L.C., Lauronen L., Beniczky S. (2023). Routine and sleep EEG: Minimum recording standards of the International Federation of Clinical Neurophysiology and the International League Against Epilepsy. Epilepsia.

[60] Windaswara R., Rochim Y.N. (2025). A Systematic Review of Diagnostic Approaches for Pediatric Epilepsy: An Evidence-Based Framework from Clinical Assessment to Precision Medicine. Int. J. Med. Sci. Health Res..

[61] Gefferie S.R., Jiménez-Jiménez D., Visser G.H., Helling R.M., Sander J.W., Balestrini S., Thijs R.D. (2023). Transcranial magnetic stimulation-evoked electroencephalography responses as biomarkers for epilepsy: A review of study design and outcomes. Hum. Brain Mapp..

[62] Ma Y. (2018). The Challenge of microRNA as a Biomarker of Epilepsy. Curr. Neuropharmacol..

[63] Oztas B., Sahin D., Kir H., Kuskay S., Ates N. (2021). Effects of leptin, ghrelin and neuropeptide y on spike-wave discharge activity and certain biochemical parameters in WAG/Rij rats with genetic absence epilepsy. J. Neuroimmunol..

[64] Chen X., Dong J., Jiao Q., Du X., Bi M., Jiang H. (2022). Sibling” battle or harmony: Crosstalk between nesfatin-1 and ghrelin. Cell. Mol. Life Sci..

[65] Keloglan S.M., Aycik F.B., Kocacan S.E., Yazgan B., Ayyildiz M., Agar E. (2023). Nesfatin 1 exerts anticonvulsant effect by reducing oxidative stress in experimental epilepsy model. Acta Neurobiol. Exp..

[66] Erfani S., Moghimi A., Aboutaleb N., Khaksari M. (2019). Protective effects of Nesfatin-1 peptide on cerebral ischemia reperfusion injury via inhibition of neuronal cell death and enhancement of antioxidant defenses. Metab. Brain Dis..

[67] Jin X., Guan K., Chen Z., Sun Y., Huo H., Wang J., Dong H. (2022). The protective effects of nesfatin-1 in neurological dysfunction after spinal cord injury by inhibiting neuroinflammation. Brain Behav..

[68] Michetti F., Clementi M.E., Di Liddo R., Valeriani F., Ria F., Rende M., Di Sante G., Spica V.R. (2023). The S100B Protein: A Multifaceted Pathogenic Factor More Than a Biomarker. Int. J. Mol. Sci..

